# Expression of Beclin Family Proteins Is Associated with Tumor Progression in Oral Cancer

**DOI:** 10.1371/journal.pone.0141308

**Published:** 2015-10-27

**Authors:** Jing-Lan Liu, Fen-Fen Chen, Shun-Fu Chang, Cheng-Nan Chen, Jrhau Lung, Cheng-Hsing Lo, Fang-Hui Lee, Ying-Chou Lu, Chien-Hui Hung

**Affiliations:** 1 Department of Pathology, Chang Gung Memorial Hospital Chiayi Branch, Chiayi, Taiwan; 2 Graduate Institute of Clinical Medical Sciences, College of Medicine, Chang Gung University, Taoyuan, Taiwan; 3 Department of Medical Research and Development, Chang Gung Memorial Hospital Chiayi Branch, Chiayi, Taiwan; 4 Department of Biochemical Science and Technology, National Chiayi University, Chiayi, Taiwan; 5 Division of Pulmonary and Critical Care Medicine, Department of Medicine, Chang Gung Memorial Hospital Chiayi Branch, Chiayi, Taiwan; 6 Department of Oral and Maxillofacial Surgery, St. Martin De Porres Hospital, Chiayi, Taiwan; 7 Department of Otolaryngology, St. Martin De Porres Hospital, Chiayi, Taiwan; University of Manitoba, CANADA

## Abstract

**Background:**

Beclin 1 and Beclin 2 are autophagy-related proteins that show similar amino acid sequences and domain structures. Beclin 1 established the first connection between autophagy and cancer. However, the role of Beclin 2 in cancer is unclear. The aims of this study were to analyze Beclin 1 and Beclin 2 expressions in oral cancer tissues and in cell lines, and to evaluate their possible roles in cancer progression.

**Methods:**

We investigated Beclin 1 and Beclin 2 expressions by immunohistochemistry in 195 cases of oral cancer. The prognostic roles of Beclin 1 and Beclin 2 were analyzed statistically. *In vitro*, overexpression and knockdown of Beclin proteins were performed on an oral cancer cell line, SAS. The immunofluorescence and autophagy flux assays confirmed that Beclin proteins were involved in autophagy. The impacts of Beclin 1 and Beclin 2 on autophagy and tumor growth were evaluated by conversion of LC3-I to LC3-II and by clonogenic assays, respectively.

**Results:**

Oral cancer tissues exhibited aberrant expressions of Beclin 1 and Beclin 2. The cytoplasmic Beclin 1 and Beclin 2 expressions were unrelated in oral cancer tissues. In survival analyses, high cytoplasmic Beclin 1 expression was associated with low disease specific survival, and negative nuclear Beclin 1 expression was associated with high recurrent free survival. Patients with either high or low cytoplasmic Beclin 2 expression had significantly lower overall survival and disease specific survival rates than those with moderate expression. In oral cancer cells, overexpression of either Beclin 1 or Beclin 2 led to autophagy activation and increased clonogenic survival; knockdown of Beclin 2 impaired autophagy and increased clonogenic survival.

**Conclusions:**

Our results indicated that distinct patterns of Beclin 1 and Beclin 2 were associated with aggressive clinical outcomes. Beclin 1 overexpression, as well as Beclin 2 overexpression and depletion, contributed to tumor growth. These findings suggest Beclin proteins are associated with tumorigenesis.

## Introduction

Oral cancer is one of the most common cancers and a growing problem worldwide. High incidence rates occur in South and Southeast Asia, parts of Europe, parts of Latin America, and Pacific regions [1]. Recently, the incidence of oral cancer has increased, particularly in economically developed countries [2]. The vast majority of oral cancer is squamous cell carcinoma, which arises from the squamous mucosa of oral surface. Despite the similar histologic phenotype, the molecular basis of oral cancer is highly complex, which exhibits multiple genetic and epigenetic mutations [3, 4]. Identifying new biomarkers for oral cancer could be applied in developing new therapeutic interventions.

Autophagy is an evolutionarily conserved catabolic process that involves the degradation of unnecessary intracellular metabolites and damaged organelles by lysosomal enzymes. The process involves the formation of autophagosomes, which engulf the unwanted cytoplasmic contents and then deliver them to lysosomes. The primary function of autophagy is to maintain cellular homeostasis, particularly in nutrient-deprived conditions. Among the autophagy-related genes, *beclin 1* established the first connection between autophagy and cancer [5], and regarded as a haploinsufficient tumor suppressor gene [6, 7]. Beclin 1 participates in autophagosome nucleation at the early step of autophagy. In addition, Beclin 1 interacts with many positive and negative regulators that affect autophagic activity and tumorigenesis [8–12]. It acts as a crucial mediator in autophagy regulation. *Beclin 2*, once called *BECN1P1*, was originally regarded as a pseudogene. In 2013, He et al exposed that the gene encodes a protein showing high similarities with Beclin 1 in amino acid sequence and domain structures, and they named the novel protein Beclin 2 [13]. This new identified protein was found to be functioned in autophagy, endolysosomal degradation, and metabolism. It also interacts with several binding partners of Beclin 1, including ATG14, AMBRA1, VPS34, and Bcl-2. These data suggested its role in regulating autophagy [13].

The association of Beclin 2 and cancer has not been addressed, and the relationship between Beclin 2 and Beclin 1 expression and the outcomes of cancer patients have never been studied. In this study, we investigated the expressions of Beclin 1 and Beclin 2 in oral cancer tissues, and examined the associations among expressions of Beclin 1 and Beclin 2, clinicopathologic features, and survival rates. We also inspected the effects of alteration of Beclin proteins on autophagy and cancer cell proliferation.

## Materials and Methods

### Patients and tissue specimens

This retrospective study was approved by the Institutional Review Board of St. Martin De Porres Hospital (Chiayi City, Taiwan) and Chang Gung Memorial Hospital Chiayi Branch (Chiayi County, Taiwan). Patient information was anonymized and de-identified prior to analysis. Formalin-fixed, paraffin-embedded tissue samples from oral cancer patients receiving surgical resection were obtained from the archives of St. Martin De Porres Hospital between January 2003 and December 2008. All of the cases were first diagnosed, histologically confirmed squamous cell carcinoma. Patients who received preoperative neoadjuvant therapy or had a tumor size of less than 5 mm were excluded from this study. Clinical information, including patients’ age, sex, tumor size, lymph node status, metastasis, and follow-up data, was obtained from medical records. The tumor-node-metastasis (TNM) stage was defined according to the seventh edition of the American Joint Committee on Cancer staging system [14]. Overall survival (OS) was defined as the time between surgery and death of all causes. Disease specific survival (DSS) was defined as the time between surgery and death caused by oral cancer. Recurrence free survival (RFS) was defined as the time between surgery and the first local or systemic recurrence.

A total of 198 cases of oral cancer were initially included. Three cases displaying limited or no tissue in the sections on tissue microarrays were excluded. The study finally consisted of 195 cases for immunohistochemical stains and statistical analysis.

### Pathologic examination

The hematoxylin and eosin-stained slides of each case were re-evaluated by a pathologist to confirm the histologic type, grade, status of necrosis and lymphovascular invasion. The histologic grade was classified as grade 1 (well differentiated), grade 2 (moderately differentiated), and grade 3 (poorly differentiated). Cases with necrotic areas constituting more than 10% of the tumor areas were regarded as “extensive necrosis,” and the other cases were recorded as “limited or no necrosis.” Lymphovascular invasion was defined as the presence of cancer cells within lymphovascular channels.

### Tissue microarray construction

Tissue microarray construction was performed as previously described [15]. In brief, a pathologist analyzed the hematoxylin and eosin-stained slides, and then marked the representative areas to be sampled on the slides and the corresponding tissue blocks. The specimen core selection from tissue blocks included two 1.5-mm-diameter cores for each case of cancer tissues, and one 1.5-mm-diameter core for each case of the normal oral mucosas. The selected cores were punched and transferred to the paraffin-recipient blocks. Consecutive 4-μm-thick sections were produced, and hematoxylin and eosin staining was performed on each recipient block to confirm the presence of the selected zones.

### Immunohistochemistry

Immunohistochemical stains were performed on the 4-μm-thick sections from the tissue microarrays by the Leica Bond-Max autostainer (Leica Microsystems, Bondbiotech, Taichung, Taiwan) according to the manufacturer’s instructions with minor modifications. The sections were deparaffinized by Bond Dewax Solution (Leica Microsystems) and rehydrated by graded alcohol. Heat-induced antigen retrieval was achieved by Bond Epitope Retrieval Solution 1 (Leica Microsystems) for 20 minutes at 100°C. The slides were incubated in hydrogen peroxide for 5 minutes to reduce endogenous peroxidase activity. The slides were then incubated for 30 minutes at room temperature with mouse monoclonal antibodies against Beclin 1 (1: 400, rabbit polyclonal, Abcam, Cambridge, UK) and Beclin 2 (1: 400, rabbit polyclonal, Novus biologicals, Littleton, CO, USA). A post-primary IgG linker reagent was applied for 8 minutes, and the slides were incubated with a polymeric horseradish peroxidase IgG reagent for 8 minutes to localize the primary antibody. Diaminobenzidine tetrahydrochloride (DAB) was used as the substrate to detect antigen-antibody binding. Finally, hematoxylin was applied for 5 minutes to counterstain nuclei.

### Assessment of immunohistochemistry

The intensity, percentage, and subcellular localization of the immunohistochemical staining of each case were recorded. Normal tonsillar tissue was used as the positive control. Staining omitting the primary antibody was performed as the negative control. Cytoplasmic and nuclear expressions were assessed separately. The intensity and percentage of positively stained cells in each core were scanned in a low power field (100 X) and then assessed in high power fields (400 X). The intensity of staining was recorded as 0, 1, 2, and 3 referring to negative, weak, moderate, and strong staining, respectively. The percentage of positive cells was recorded from 0% to 100%. The results of staining were scored using quick (Q) score, which was obtained by multiplying the percentage of positive cells (P) by the intensity (I) (Q = P × I; maximum = 300) [16]. Two pathologists independently evaluated the results of immunohistochemical staining without knowledge of clinicopathologic data. Conflicting results were resolved by a multihead microscope.

### Cutoff point selection for immunohistochemistry

For cytoplasmic expressions of Beclin 1 and Beclin 2, the optimal cutoff points of the Q scores were determined by X-Tile software 3.6.1, as previously described [17]. The program calculated the chi-square values at all possible divisions based on the log-rank test for Kaplan-Meier estimates. For cytoplasmic expression of Beclin 1, one optimal point with the highest value was determined. The cutoff point of Q scores with highest chi-square values were 55, 110, and 25 for OS, DSS, and RFS, respectively. We selected 63 as cutoff Q score for Beclin 1 cytoplasmic expression. For cytoplasmic expression of Beclin 2, the chi-square values determined by 2 optimal cutoff points were greater than those determined by 1 optimal point. Thus we selected 2 cutoff points to classify its expression into 3 groups: low, moderate, and high. The cutoff points of Q score with the highest chi-square values were (35, 125), (35, 145), and (25, 85) for OS, DSS, and RFS, respectively. We selected (32, 118) as cutoff Q scores for Beclin 2 cytoplasmic expression.

Only a minority of cases showed nuclear expression of Beclin 1 or Beclin 2. Thus, cases with more than 10% areas of nuclear staining were classified as positive; the others were classified as negative.

### Cell cultures and reagents

The human oral squamous cell carcinoma cell line SAS, established from a poorly differentiated squamous cell carcinoma of the tongue, was cultured in 10% FBS supplemented DMEM. Plasmids expressing flag-tagged Beclin 1 and Beclin 2 were obtained from Addgene and a gift from Levine’s lab, respectively. To generate a lentiviral vector expressing Beclin 2, the PCR fragment of flag-tagged *beclin 2* was cloned into pLX301 lentiviral vector (Addgene). Lentiviruses were generated by cotransfecting HEK293 cells with plasmids pLX301-Beclin 2, pCMVDR8.2 and pMD.G by lipofectamine 2000. Lentiviruses released to culture medium were collected at 48 h and 72 h after transfection. SAS cells stably overexpressing Beclin 2 were established by infecting lentivirus expressing Beclin *2* and puromycin selection. Bafilomycin, an autophagy inhibitor preventing fusion between autophagosomes and lysosomes, was purchase from Sigma-Aldrich (St Louis, MO, USA).

### siRNA knockdown

Double-stranded small interfering RNA (siRNA) against *Atg5* and *beclin 2* were purchase from Invitrogen (Carlsbad, CA, USA) and Santa cruz Biotechnology (Santa Cruz, CA, USA), respectively. SAS cells were seeded in 6 well plates, and transfected with 100 pmole siRNA by RNAiMax (Invitrogen) at the following day.

### Immunoblot analysis

Cells were lysed in PBS that contained 1% Triton X-100 and protease inhibitor cocktail (Sigma-Aldrich). The lysates were then centrifuged at 12,000x g at 4°C for 15 min. The proteins in the lysate were separated by SDS-PAGE, transferred to a polyvinylidene difluoride membrane and detected by immunoblotting. Anti-Flag and Anti-Atg*5* were purchased from Sigma-Aldrich. Anti-LC3 was obtained from Nanotool (Teningen, Germany). Anti-Beclin 1 and Anti-Beclin 2 were purchased from Santa cruz Biotechnology and Novus Biologicals, respectively. Anti-p62 was purchased from Abcam.

### Immunofluorescence staining

Cells were fixed by 4% paraformaldehyde, and processed for immunofluorescence staining with primary antibody against anti-Flag (M2) and incubation with fluorescence-labeled secondary antibody (Invitrogen). Finally, cells were observed and images were captured under Leica SP5 fluorescence microscope.

### Clonogenic assay

SAS cells transfected with plasmids or siRNA were plated at about 200 cells per well in 6-well plates and cultured for 10 days. Cells were washed with PBS and stained with 1% crystal violet in methanol for 15 min at room temperature. After washing out the dye, colonies were counted.

### Statistical analysis

The chi-square tests were used to evaluate the association of expression of Beclin proteins and clinicopathologic features. The correlations of Q scores were assessed by Spearman rank correlation tests, and the Spearman’s rho coefficients were illustrated as heat map. Curves for OS, DSS, and RFS were drawn using the Kaplan-Meier method, and the differences in survival rates were compared by log-rank tests. The univariate and multivariate Cox proportion regression models were used to estimate the hazard ratios and 95% confidence intervals for patients’ outcomes. All *P* values were 2-sided. A *P* value less than .05 was considered statistically significant. The colony numbers in clonogenic assays were represented as ratios of control +/- standard errors. Statistical analysis was performed using SPSS version 19.0 software (SPSS, Inc., Armonk, NY, USA) and Excel 2013 (Microsoft Excel, Microsoft, Redmond, WA, USA) for Windows.

## Results

### Aberrant Beclin 1 and Beclin 2 expressions in oral cancer tissues

**Fig 1:**
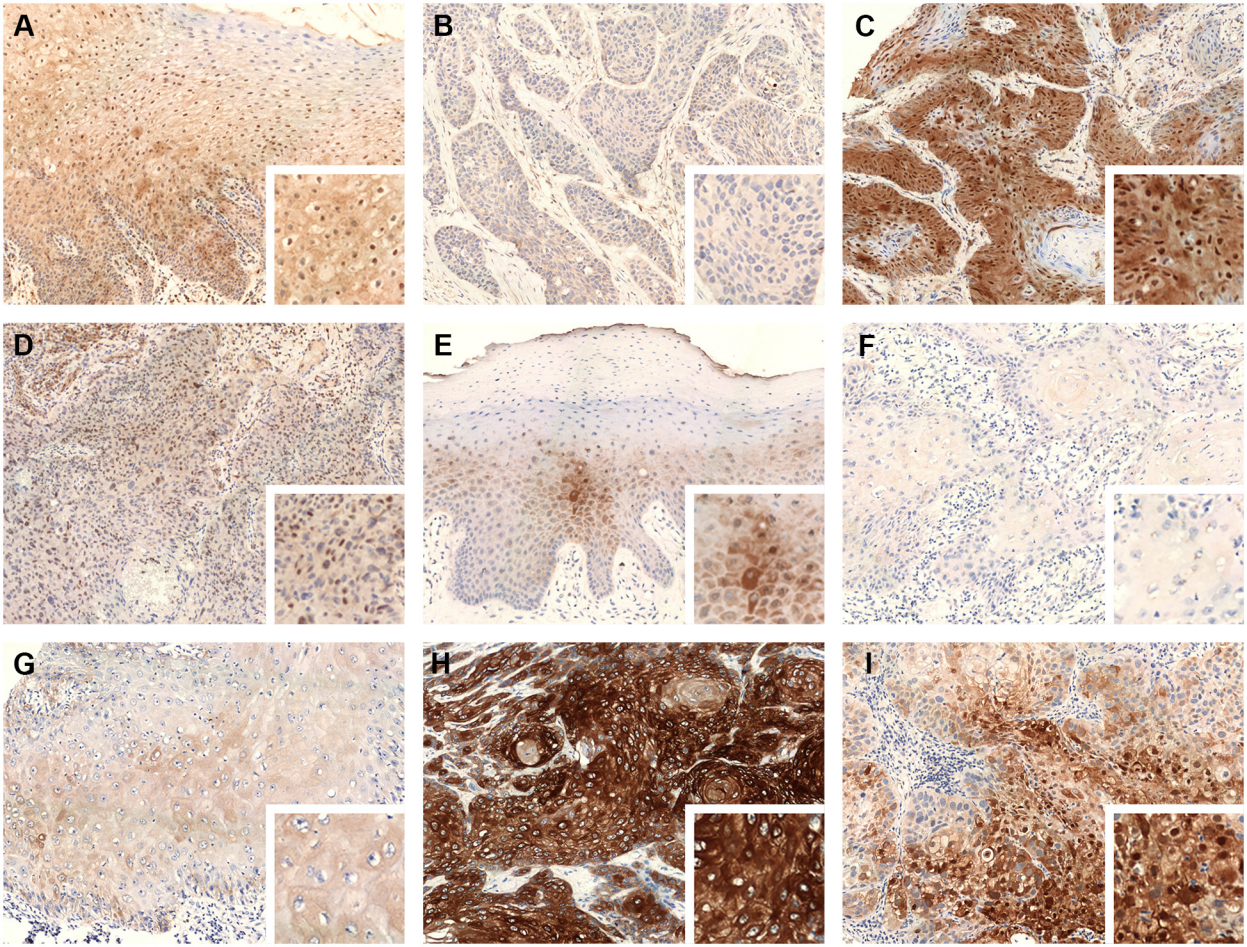
Representative cases of immunohistochemical stain of Beclin proteins (original magnification x 200, right lower inset: original magnification x 400). (A) Weak to moderate cytoplasmic and nuclear expression of Beclin 1 in normal oral squamous mucosa. (B) Low cytoplasmic expression of Beclin 1 in an oral cancer tissue. (C) High cytoplasmic and positive nuclear expression of Beclin 1 in an oral cancer tissue. (D) Low cytoplasmic and positive nuclear expression of Beclin 1 in an oral cancer tissue. (E) Weak to moderate cytoplasmic expression of Beclin 2 in normal oral squamous mucosa. (F) Low cytoplasmic expression of Beclin 2 in an oral cancer tissue. (G) Moderate cytoplasmic expression of Beclin 2 in an oral cancer tissue. (H) High cytoplasmic expression of Beclin 2 in an oral cancer tissue. (I) Moderate cytoplasmic and positive nuclear expression of Beclin 2 in an oral cancer tissue.

To evaluate Beclin 1 and Beclin 2 expressions and their subcellular localization, we performed immunohistochemistry on 195 cases of oral cancer tissues and normal oral mucosas. Beclin 1 and Beclin 2 expressions were primarily located in the cytoplasm, and occasionally in the nuclei. The normal oral mucosas showed weak to moderate cytoplasmic expression and focal nuclear expression of Beclin 1 and Beclin 2 (Fig 1A and 1E). The oral cancer tissues exhibited variable cytoplasmic expressions of Beclin 1 and Beclin 2, ranging from total loss to diffuse strong expression (Fig 1B–1D and 1F–1H). The mean Q scores of cytoplasmic Beclin 1 and Beclin 2 were 67.46 ± 55.19 and 75.26 ± 62.16, respectively. The cutoff points for Beclin 1 and Beclin 2 were described in materials and methods. For cytoplasmic Beclin 1, 111 cases (56.92%) were classified as low expression, and 84 cases (43.08%) were classified as high expression. For cytoplasmic Beclin 2, 65 patients (33.33%) were classified as low expression; 83 patients (42.56%) were classified as moderate expression; 47 patients (24.10%) were classified as high expression. Only a small subset of oral cancer tissues expresses nuclear staining of Beclin 1 (33/195, 16.92%) and Beclin 2 (29/195, 14.87%) (Fig 1C, 1D and 1I).

### Associations of Beclin 1 and Beclin 2 expressions with clinicopathologic characteristics and the autophagosomal marker in oral cancer tissues

We compared Beclin 1 and Beclin 2 expressions with age, sex, tumor size, lymph node status, metastasis, stage, grade, lymphovascular invasion, and necrosis (Table 1). Beclin 1 expression was unrelated to clinicopathologic features. Low cytoplasmic expression of Beclin 2 was associated with higher histologic grade (a poor prognostic factor) compared to moderate and high expressions. Positive nuclear expression of Beclin 2 was connected to poor prognostic factors, including lymph node metastasis and lymphovascular invasion.

**Table 1:** Relationships between Beclin 1 and Beclin 2 expressions and clinicopathologic variables in 195 cases of oral cancer.

	Cytoplasmic Beclin 1		*P* value	Nuclear Beclin 1		*P* value	Cytoplasmic Beclin 2			*P* value	Nuclear Beclin 2		*P* value
Variables	Low	High		Negative	Positive		Low	Moderate	High		Negative	Positive	
Age													
≦ 50 years	41	30	0.861	59	12	0.995	29	26	16	0.234	63	8	0.284
> 50 years	70	54		103	21		36	57	31		103	21	
Sex													
Male	104	83	0.141^a^	157	30	0.136^a^	62	78	47	0.243	159	28	0.847^a^
Female	7	1		5	3		3	5	0		7	1	
Tumor size													
T1/T2	74	52	0.491	108	18	0.184	46	52	28	0.419	106	20	0.595
T3/T4	37	32		54	15		19	31	19		60	9	
Lymph node status													
Negative	81	64	0.610	121	24	0.814	49	63	33	0.754	128	17	0.035*
Positive	30	20		41	9		16	20	14		38	12	
Metastasis													
Negative	109	83	1.000^a^	160	32	0.428^a^	63	82	47	0.404	164	28	0.385^a^
Positive	2	1		2	1		2	1	0		2	1	
Stage													
I/II	61	45	0.848	91	15	0.260	38	45	23	0.607	92	14	0.476
III/IV	50	39		71	18		27	38	24		74	15	
Grade													
G1/G2	94	73	0.662	139	28	1.000^a^	48	75	44	0.004*	143	24	0.576^a^
G3	17	11		23	5		17	8	3		23	5	
Lymphovascular invasion													
Absent	84	63	0.914	122	25	0.956	51	62	34	0.746	130	17	0.023*
Present	27	21		40	8		14	21	13		36	12	
Necrosis													
Limited/ no	82	63	0.858	121	24	0.814	44	66	35	0.263	123	22	0.841
Extensive	29	21		41	9		21	17	12		43	7	

*P < 0.05

^a^Fisher exact test

**Fig 2:**
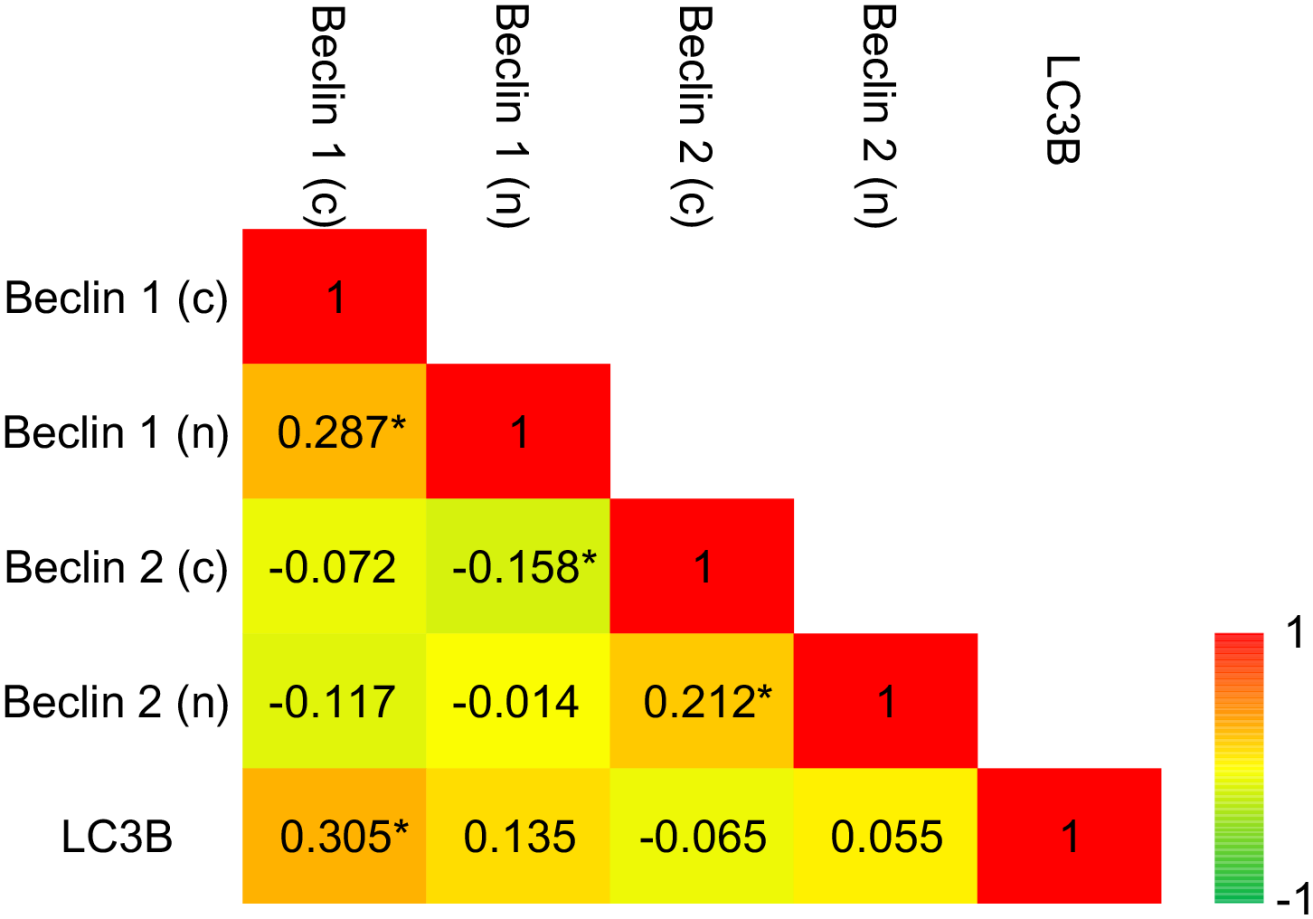
Color-coded correlation matrix for expressions of Beclin proteins and LC3B. Color scale for Spearman’s coefficient rho is given in the lower right, with green representing a negative correlation and red positive. *P < 0.05.

The nuclear and cytoplasmic expressions of Beclin 1 showed weak correlation (Spearman’s rho = 0.287, *P* < 0.001). The nuclear and cytoplasmic expressions of Beclin 2 were also weakly correlated (Spearman’s rho = 0.212, *P* = 0.003). The cytoplasmic expression of Beclin 1 and Beclin 2 was unrelated (Spearman’s rho = -0.072, *P* = 0.318). The relationships of Beclin proteins and the autophagosomal marker, LC3B, were also evaluated. The autophagosomes represented by LC3B punctae were evaluated in our previous study [18]. Only cytoplasmic Beclin 1 correlated to the level of LC3B punctae (Spearman’s rho = 0.305, *P* < 0.001) (Fig 2).

### Distinct expression patterns of Beclin proteins are associated with poor prognosis

**Fig 3:**
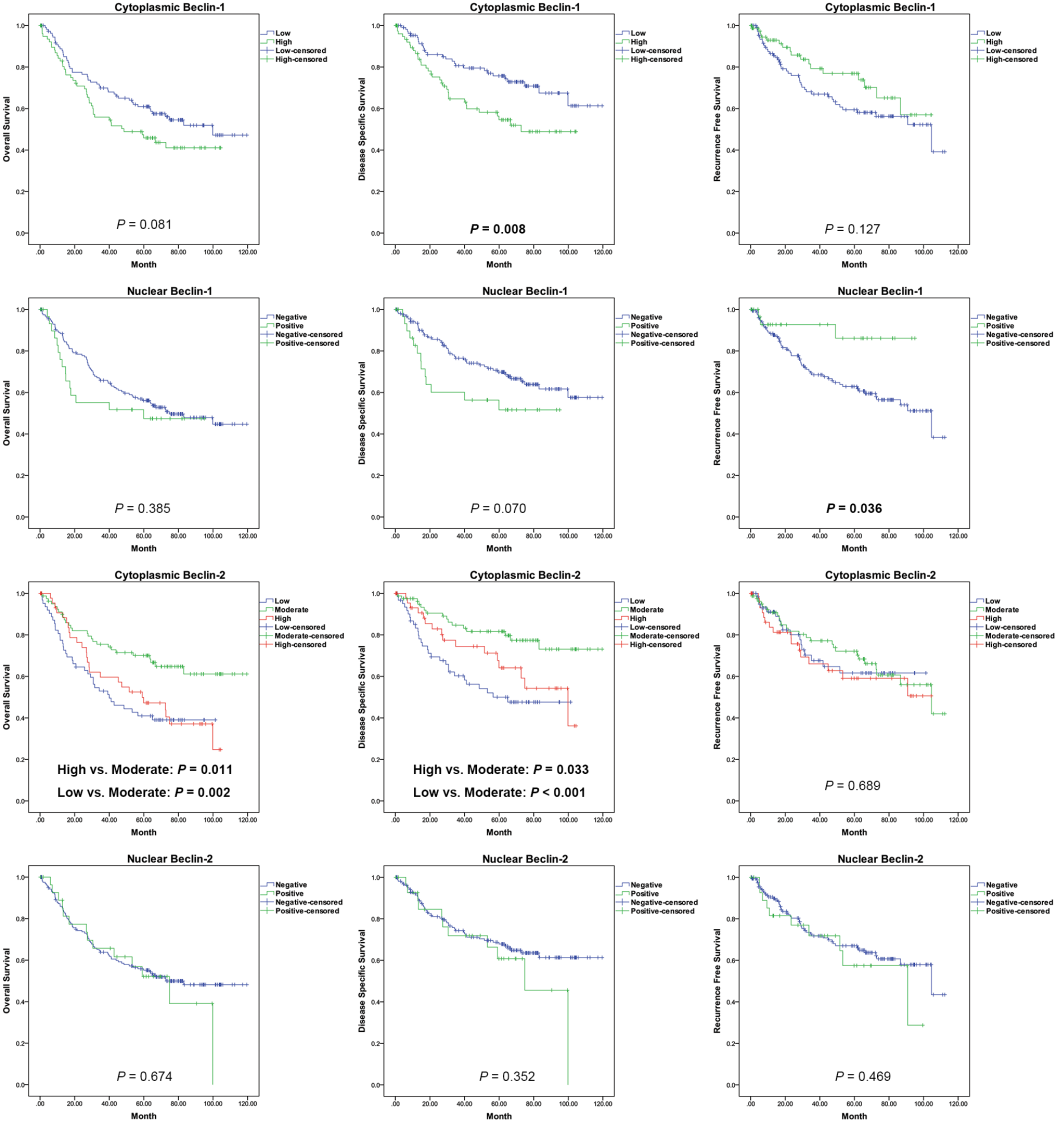
The Kaplan-Meier curves for OS, DSS and RFS rates according to cytoplasmic and nuclear Beclin 1 and Beclin 2 expression in oral cancers.

The Kaplan-Meier curves of cumulative OS, DSS, and RFS probabilities for Beclin 1 and Beclin 2 are shown in Fig 3. Patients with high cytoplasmic Beclin 1 expression showed low DSS (*P* = 0.008), and those with negative nuclear Beclin 1 expression showed high RFS (*P* = 0.036). Patients with either high or low cytoplasmic Beclin 2 expression had significantly lower OS and DSS rates compared with patients with moderate cytoplasmic Beclin 2 expression. The survival probabilities between patients with low and high cytoplasmic expressions were not statistically significant. The nuclear Beclin 2 expression was unassociated with survival status.

The results of univariate and multivariate survival analyses using the Cox proportional hazard regression methods are shown in Tables 2, 3 and 4. High cytoplasmic Beclin 1 and negative of nuclear Beclin 1 were associated with poor DSS and RFS under univariate and multivariate analyses, respectively. Additionally, cytoplasmic Beclin 2 expression was an independent prognostic factor for OS and DSS. Low and high cytoplasmic Beclin 2 expressions were associated with aggressive clinical outcomes.

**Table 2:** Univariate and multivariate analyses of OS in 195 cases of oral cancer.

	Univariate analysis				Multivariate analysis			
	HR	95% CI		*P* value	HR	95% CI		*P* value
Variable		Low	High			Low	High	
Age (> 50 vs. ≦ 50 years)	1.216	0.781	1.892	0.387				
Sex (Male vs. Female)	2.295	0.565	9.325	0.246				
Stage (III/IV vs. I/II)	3.013	1.953	4.646	< 0.001*	2.019	1.156	3.525	0.013*
Grade (G3 vs. G1/G2)	1.328	0.737	2.393	0.344				
Lymphovascular invasion (Present vs. Absent)	3.399	2.211	5.225	< 0.001*	2.406	1.416	4.088	0.001*
Necrosis (Extensive vs. Limited/no)	1.828	1.182	2.825	0.007*	1.272	0.798	2.028	0.313
Cytoplasmic Beclin 1 (High vs. Low)	1.443	0.953	2.186	0.083				
Nuclear Beclin 1 (Positive vs. Negative)	1.278	0.734	2.228	0.386				
Cytoplasmic Beclin 2								
Low vs. Moderate	2.253	1.369	3.709	0.001*	2.698	1.614	4.509	< 0.001*
High vs. Moderate	1.975	1.152	3.388	0.013*	1.865	1.084	3.207	0.024*
Nuclear Beclin 2 (Positive vs. Negative)	1.130	0.639	2.001	0.674				

OS, overall survival; CI, confidence interval.

* *P*< 0.05

**Table 3:** Univariate and multivariate analyses of DSS in 195 cases of oral cancer.

	Univariate analysis				Multivariate analysis			
	HR	95% CI		*P* value	HR	95% CI		*P* value
Variable		Low	High			Low	High	
Age (> 50 vs. ≦ 50 years)	1.097	0.647	1.861	0.731				
Sex (Male vs. Female)	3.090	0.428	22.308	0.263				
Stage (III/IV vs. I/II)	5.608	3.082	10.205	< 0.001*	4.161	2.086	8.299	< 0.001*
Grade (G3 vs. G1/G2)	1.367	0.673	2.777	0.387				
Lymphovascular invasion (Present vs. Absent)	3.399	2.211	5.225	< 0.001*	2.599	1.430	4.726	0.002*
Necrosis (Extensive vs. Limited/no)	1.692	0.990	2.891	0.054				
Cytoplasmic Beclin-1 (High vs. Low)	1.955	1.180	3.238	0.009*	1.706	1.021	2.850	0.041*
Nuclear Beclin-1 (Positive vs. Negative)	1.751	0.947	3.239	0.074				
Cytoplasmic Beclin-2								
Low vs. Moderate	3.024	1.635	5.590	< 0.001*	3.984	2.115	7.502	< 0.001*
High vs. Moderate	2.065	1.031	4.134	0.041*	1.722	0.853	3.480	0.130
Nuclear Beclin-2 (Positive vs. Negative)	1.363	0.708	2.621	0.354				

DSS, disease specific survival; CI, confidence interval.

* *P*< 0.05

**Table 4:** Univariate and multivariate analyses of RFS in 195 cases of oral cancer.

	Univariate analysis				Multivariate analysis			
	HR	95% CI		*P* value	HR	95% CI		*P* value
Variable		Low	High			Low	High	
Age (> 50 vs. ≦ 50 years)	0.858	0.508	1.449	0.567				
Sex (Male vs. Female)	1.037	0.324	3.320	0.951				
Stage (III/IV vs. I/II)	1.699	1.017	2.838	0.043*	1.195	0.616	2.318	0.598
Grade (G3 vs. G1/G2)	0.835	0.358	1.945	0.676				
Lymphovascular invasion (Present vs. Abscent)	2.557	1.460	4.480	0.001*	2.272	1.104	4.676	0.026*
Necrosis (Extensive vs. Limited/no)	1.202	0.676	2.134	0.531				
Cytoplasmic Beclin-1 (High vs. Low)	0.646	0.367	1.138	0.130				
Nuclear Beclin-1 (Positive vs. Negative)	0.308	0.096	0.986	0.047*	0.300	0.094	0.961	0.043*
Cytoplasmic Beclin-2								
Low vs. Moderate	1.102	0.594	2.044	0.759				
High vs. Moderate	1.318	0.702	2.472	0.390				
Nuclear Beclin-2 (Positive vs. Negative)	1.286	0.650	2.547	0.470				

RFS, recurrence free survival; CI, confidence interval.

* *P*< 0.05.

### Either Beclin 1 or Beclin 2 overexpression induces autophagy and promotes oral cancer cell growth

**Fig 4:**
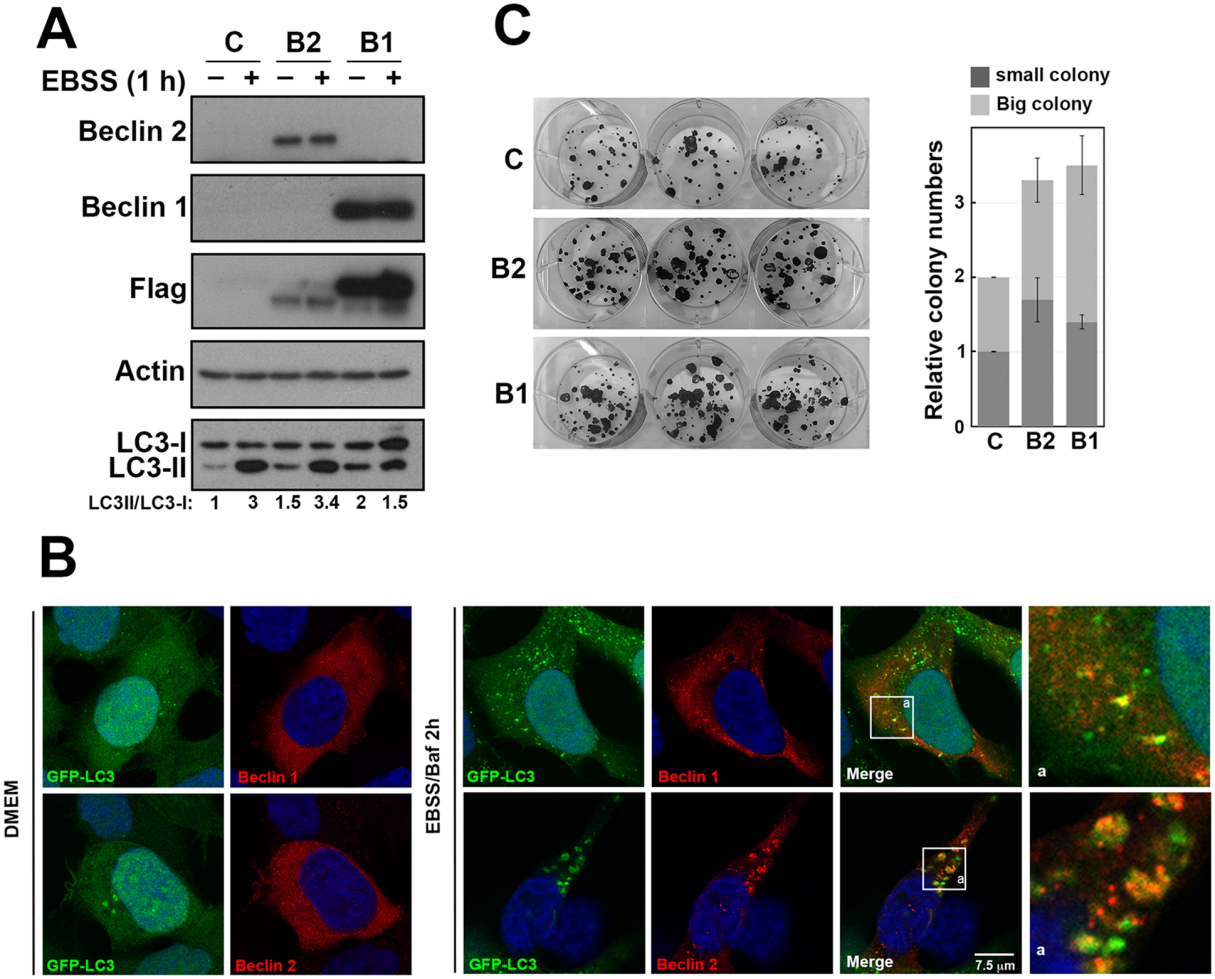
The effects of overexpressing Beclin 1 and Beclin 2 in autophagy and cell survival. (A) SAS cells were transfected with control plasmid, plasmids expressing flag-tagged Beclin 1 (B1) or Beclin 2 (B2). At 24 h posttransfection, cells were treated with Earle’s balanced salt solution (EBSS) for 1 h starvation. Lysate were prepared and analyzed by immunoblot using the indicated antibodies. The intensity of LC3-I and LC3-II was measured using ImageJ and the ratio of LC3-II/LC3-I are shown below. (B) HEK293 cells stably expressing GFP-LC3 were transfected with plasmid expressing flag-tagged Beclin 1 or Beclin 2 and cultured in either normal culture medium (DMEM) or starvation condition (EBSS) as indicated. Cells were fixed at 48 h after transfection. Indirect immunofluorescence staining was performed using anti-Flag M2. 4’,6-Diamidino-2-phenylindole (DAPI) staining revealed the nucleus. The box region is enlarged (a). (C) SAS cells were transfected with control or flag-tagged Beclin 1 and Beclin 2 for 24 h. Transfected cells were replated at 200 cells per well in 6-well plate. After 14 days, colonies were visualized using 1% crystal violet in methanol. The experiments were conducted three times independently.

To investigate the effects of Beclin 1 and Beclin 2 overexpression in oral cancer, we used SAS cells transfected with plasmid expressing flag-tagged Beclin 1 and Beclin 2, respectively. Overexpression of Beclin 1 or Beclin 2 led to increase of the conversion of LC3-I to LC3-II under normal condition. However, this phenomenon was inevident under starvation condition (Fig 4A). The ratio of LC3-II/LC3-I and p62 level were both increased in cells overexpressing Beclin 1 or Beclin 2 compared to control after Bafilomycin treatment in starvation (S1 Fig). These findings confirm the role of Beclin 2 in autophagy pathway.

To evaluate the subcellular localization of Beclin proteins, HEK293 cells were transfected with plasmid expressing flag-tagged Beclin 1 and Beclin 2, respectively. Under normal condition, the number of GFP-LC3 puncta was low, and Beclin 1 and Beclin 2 were mostly localized in the cytoplasm (Fig 4B, left panel). After autophagy induction by nutrient starvation and Bafilomycin treatment, cells showed increased LC3 punctae; Beclin 1 and Beclin 2 showed partial colocalization with LC3 punctae (Fig 4B, right panel). In addition, we observed that LC3 punctae were enlarged in Beclin 2 overexpressing cells compared to control and Beclin 1 overexpressing cells. Furthermore, cells with either Beclin 1 or Beclin 2 overexpression led to increased clonogenic survival ability compare to control transfected cells (Fig 4C). These findings suggest that overexpression of Beclin 1 or Beclin 2 activates autophagy and promotes cancer cell growth, and the effects of overexpression of Beclin 1 and Beclin 2 were independent from each other.

### Beclin 2 depletion impairs autophagy and promotes oral cancer cell growth

**Fig 5:**
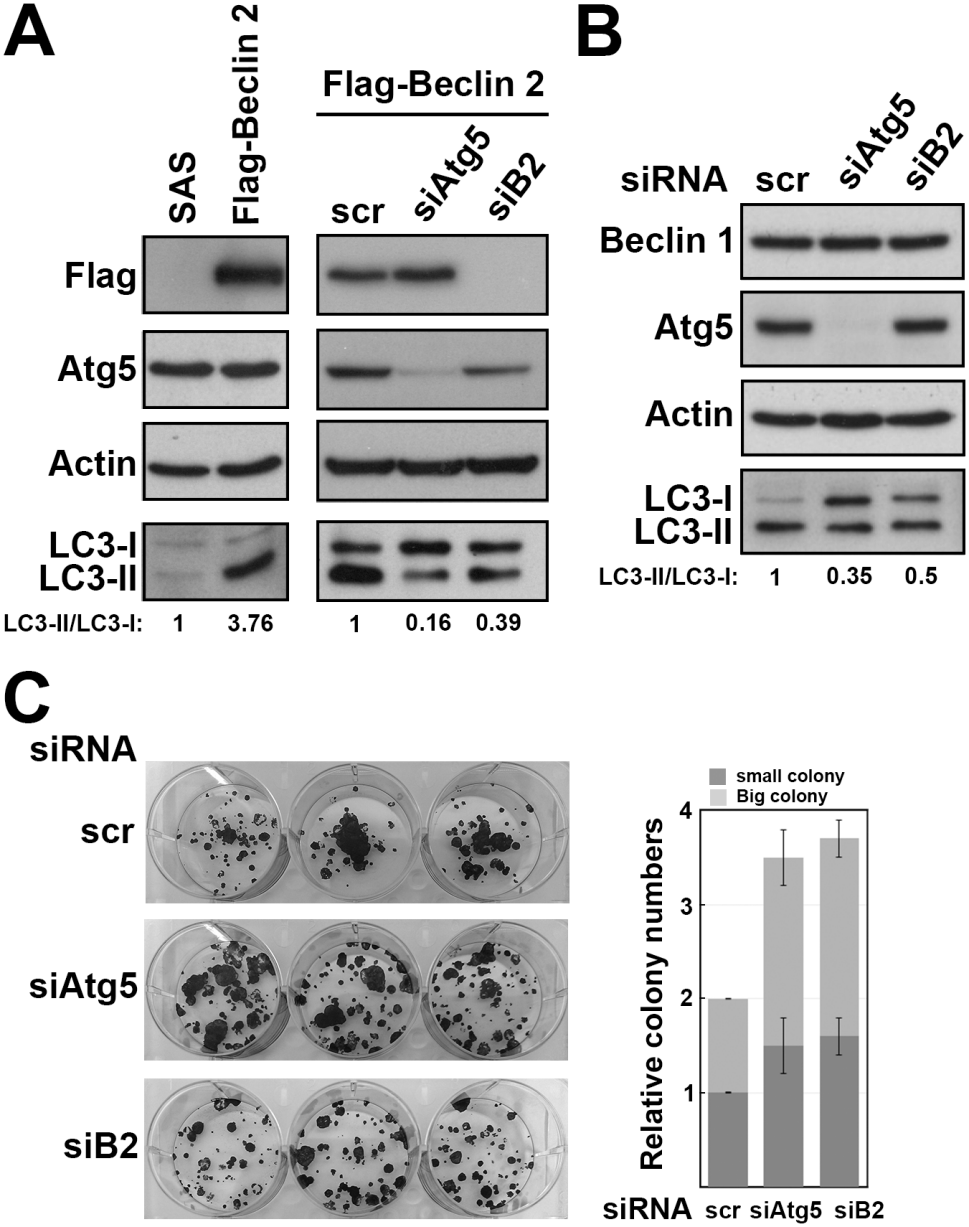
The effects of Beclin 2 knockdown in autophagy and cell survival. (A) The SAS cells or cells stably expressing flag-tagged Beclin 2 or (B) SAS cells transfected with control siRNA (scr), Atg5 siRNA (siAtg5) or Berlin 2 siRNA (siB2). Cell lysate were prepared after 48 h transfection and analyzed by immunoblot using the indicated antibodies. (C) SAS cells were transfected with control siRNA (scr), Atg5 siRNA (siAtg5) or Berlin 2 siRNA (siB2) for 24 h. Transfected cells were replated at 200 cells per well in 6-well plate. After 14 days, colonies were visualized using 1% crystal violet in methanol. The experiments were conducted three times independently.

To inspect the effect of Beclin 2 depletion and its relationship with autophagy in oral cancer, we transfected siRNAs targeting either *beclin 2* or *Atg5* in SAS cells. Because the endogenous Beclin 2 level was low in SAS cells, the efficiency of *beclin 2* knockdown by siRNA was first determined in cells stably expressed flag-tagged Beclin 2. Cells stably expressing Beclin 2 showed higher ratio of LC3-II/LC3-I (Fig 5A, left panel). After transfecting siRNA targeting *beclin 2* or *Atg5*, expression of Beclin 2 or Atg5 was significantly reduced, and the ratios of LC3-II/LC3-I were decreased simultaneously (Fig 5A, right panel). Although the endogenous Beclin 2 was unable to detect after transfecting siRNA targeting *beclin 2*, the Beclin 1 and Atg5 levels were unaffected, and the ratio of LC3-II/LC3-I were decreased (Fig 5B). These data imply that Beclin 2 depletion causes autophagy impairment. Cells with either Beclin 2 or Atg5 depletion showed clonogenic survival ability compare to the cells treated with control siRNA (Fig 5C). These findings suggest Beclin 2 depletion impairs part of autophagy activity, and promotes cancer cell growth.

## Discussion

The Beclin family has 2 identified members, Beclin 1 and Beclin 2. The prognostic role of Beclin 1 has been widely studied in various types of human cancers, and the patients’ outcomes determined by cytoplasmic Beclin 1 expression may be specific to the cancer type. For example, low expression of Beclin 1 associated with poor prognosis in breast cancer [19, 20], ovarian cancer [21], lung cancer [22], hepatocellular carcinoma [23], cholangiocarcinoma [24], pancreatic cancer [25], hypopharyngeal cancer [26], laryngeal cancer [27], esophageal cancer [28], duodenal cancer [29], gastric cancer [30], lymphomas [31–33], etc. High expression of Beclin 1 connected to tumor aggressiveness in nasopharyngeal carcinoma [34], endometrial cancer [35], and colorectal cancer [36]. The investigations of Beclin 1 in oral cancer tissues showed that high expression was related to poor prognostic factors and/or aggressive clinical outcomes [37, 38]. Our study was consistent with previous researches, and we further revealed that overexpression of Beclin 1 in SAS induced tumor growth in clonogenic assay, demonstrating its role of tumor progression in oral cancer.

In this study, we first evaluated the expression and prognostic role of Beclin 2 in human cancer. We disclosed that oral cancer patients with either high or low expression of Beclin 2 showed poorer prognosis than those with moderate expression. The expression of Beclin 2 was independent of cancer stage (Table 1). Either high or low Beclin 2 expression in cancer tissues suggested dysfunction in autophagy and metabolism. Beclin 2 functional analyses further showed either its depletion or overexpression promoted tumor growth in the SAS oral cancer cells. These findings imply Beclin 2 is associated with tumorigenesis in oral cancer. Manipulation of gene expression of *beclin 1* did not affect Beclin 2 protein, and vice versa. The cytoplasmic expressions of the 2 proteins were also unrelated in oral cancer tissues (Fig 2). The results suggest the molecular mechanism involved in oral tumorgenesis by these 2 proteins is independent.

Autophagy has been implicated in both tumor suppression and tumor progression. Autophagy inhibits malignant transformation of normal cells by maintaining cellular homeostasis, normal metabolism, and genomic stability [39]. On established tumors, autophagy often promotes tumor cell growth by sustaining metabolism in stressful condition [40, 41]. However, the actual influence of autophagy on tumorigenesis may be context-dependent. Autophagy may regulate cell growth differently according to tissue types and specific genetic settings [42, 43]. The associations between autophagy and Beclin proteins have been well documented. Beclin proteins interact with components in class III phosphoinositide 3-kinase complex, which is required for initiation of autophagy [13, 44]. Beclin 1 is involved in the formation of phagophore (the precursor of the autophagosome) in the early step of autophagy, and is colocalized with LC3 punctae [45, 46]. Our data first demonstrated the colocalization of Beclin 2 and LC3, confirming that Beclin 2 is involved in autophagy. Although we found that manipulations of Beclin 1 or Beclin 2 level did lead to change in autophagy, their effects on tumor growth may not entirely directly due to autophagy. Beclin 1 was initially to be found to have tumor suppressor function [5]. Paradoxically, high expression of Beclin 1 was associated with tumor progression in some cancers, as previous described [34–38]. Allelic loss of the *beclin 1* inhibited tumor formation in certain genotypes of mice [47, 48]. Beclin 1 not only interacts with positive and negative regulators of autophagy, but also participates in several non-autophagic processes [49]. The impacts of Beclin 1 on tumor progression may be at least partly independent from autophagy. Furthermore, our study first established the connection of Beclin 2 and tumor progression. The relations of Beclin 2 and tumorigenesis may be more complex. The tumor progression caused by Beclin 2 overexpression and depletion may be due to different mechanisms. Our data and previous literature found that knockdown of Beclin 2 impaired but not entirely abolished autophagy [13]. The tumor growth caused by Beclin 2 depletion may be evoked by autophagy and/or autophagy-independent mechanisms. Beclin 2 shares several interacting partners of Beclin 1, and additionally required for degradation of GASP1-regulated G protein-coupled receptors (GPCRs) [13]. Many GPCRs are overexpressed in cancers and contribute to cancer cell proliferation [50]. Loss of Beclin 2 may cause accumulation of some GPCRs and lead to tumor progression. In tissues, high expression of Beclin 2 or Beclin 1, which are involved in early steps of autophagy, could be resulted from either autophagic activation or late step impairment. These may also explain why patients with either high or low Beclin 2 expression had poorer prognosis than those with moderate expression. More studies are required to address the biological mechanisms and related signaling pathways of Beclin proteins in tumorigenesis.

Most of Beclin 1 localized in the intracytoplasmic organelles, including endoplasmic reticulum, mitochondria, and perinuclear membrane. Its nuclear expression was rarely mentioned. Koukourakis et al found that high nuclear expression of Beclin 1 was associated with aggressive pathologic features in colorectal cancer [51]. Our previous study disclosed that loss of Beclin 1 nuclear positivity in malignant canine mammary tumors was associated with poor prognostic factors [52]. In this study, only a minority of oral cancer tissues showed nuclear expression of Beclin 1 or Beclin 2. We found that negativity of Beclin 1 was associated with poor recurrence free survival, yet positivity of Beclin 2 was linked to poor prognostic factors, including positive lymphovascular status and lymph node metastasis. Beclin 1 comprises a leucine-rich nuclear export signal, and inhibition of its nuclear export may impaired autophagy [53]. The leucine-rich nuclear export signal of human Beclin 1 at amino acids 180–189 is preserved in Beclin 2. Our data showed the nuclear expression of Beclin proteins was often accompanied with cytoplasmic expression in oral cancer tissues. So the nuclear expression of Beclin proteins was probably not due to damaged nuclear export activity. Moreover, the Beclin 1 or Beclin 2 nuclear expression was unrelated to autophagosomal marker (Fig 2), which implies that the Beclin proteins play some non-autophagic roles in the nucleus. Further studies are needed to discern the regulation of nuclear export and the functions in the nucleus of Beclin proteins.

In conclusion, we determined that expressions of Beclin proteins are divergent in oral cancer. Overexpression of Beclin 1 resulted in tumor progression in oral cancer, and was associated with poor prognosis. Patients with high or low expression of Beclin 2 was related to poorer clinical outcomes than those with moderate expression. Either overexpression or depletion of Beclin 2 promoted tumor cell growth. Our results suggest that Beclin proteins may be associated with tumor progression and could be potential therapeutic targets in the future.

## Supporting Information

S1 Fig The autophagy flux assays in SAS cells transfected with control plasmid, plasmids expressing flag-tagged Beclin 1 or Beclin 2. Lysate were prepared and analyzed by immunoblot using the indicated antibodies. The intensity of LC3-I and LC3-II was measured using ImageJ and the ratio of LC3-II/LC3-I are shown below.
(PDF)
