# Supplementary figures and images for: Expression of Beclin Family Proteins Is Associated with Tumor Progression in Oral Cancer
Source: PLoS One. 2015 Oct 27;10(10):e0141308. doi: 10.1371/journal.pone.0141308 (PMC4624707; doi:10.1371/journal.pone.0141308)

# S1 Fig. The autophagy flux assay.

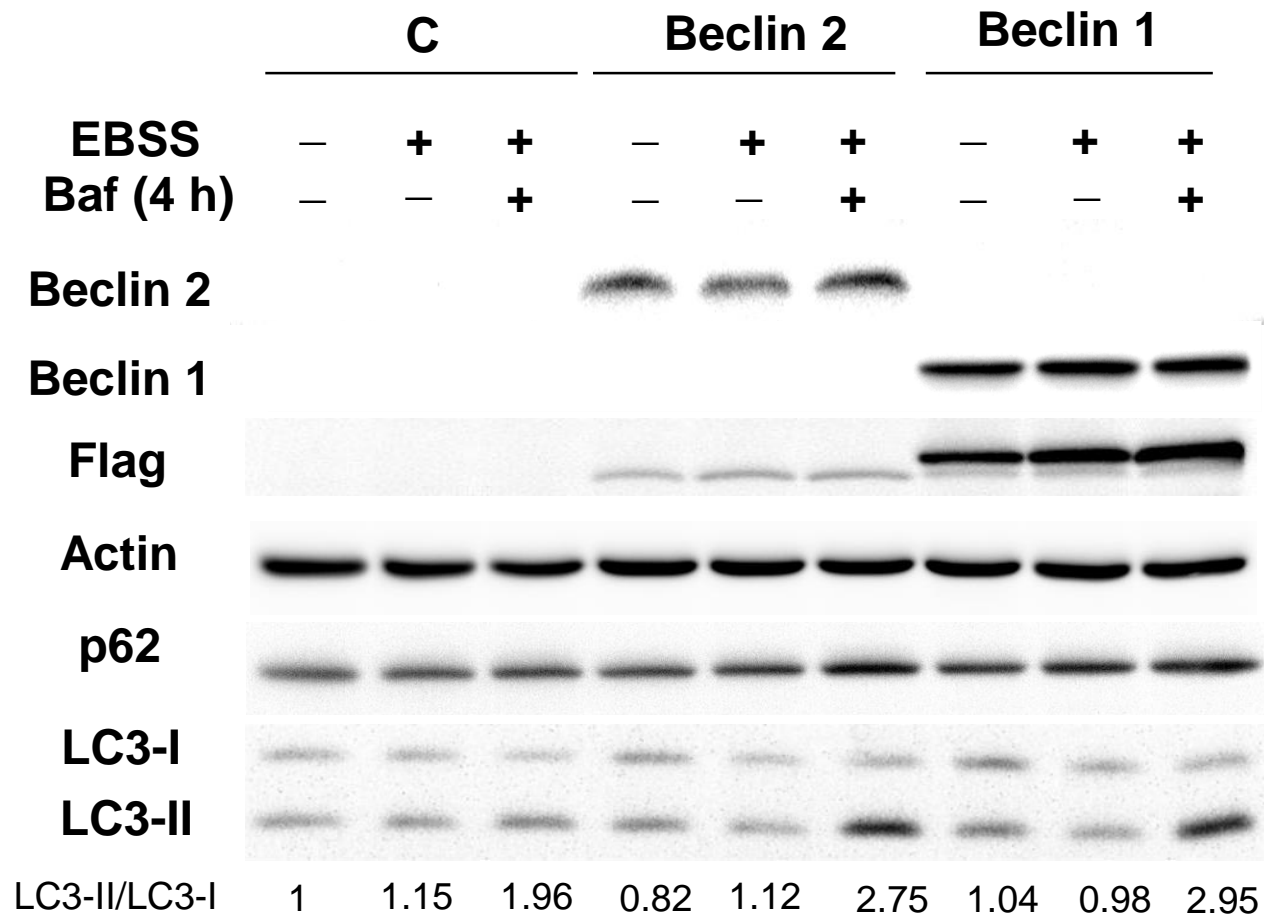

Supplement: S1 Fig — Lysate were prepared and analyzed by immunoblot using the indicated antibodies. The intensity of LC3-I and LC3-II was measured using ImageJ and the ratio of LC3-II/LC3-I are shown below. (PDF) [file pone.0141308.s001.pdf]
